# Molecular-Genetic Basis of Plant Breeding

**DOI:** 10.3390/biom12101392

**Published:** 2022-09-29

**Authors:** Elena Khlestkina, Yuri Shavrukov

**Affiliations:** 1 N.I. Vavilov All-Russian Institute of Plant Genetic Resources (VIR), Bolshaya Morskaya 42-44, 190000 St.-Petersburg, Russia; 2 College of Science and Engineering, Biological Sciences, Flinders University, Adelaide, SA 5042, Australia

Traditional plant breeding can be improved significantly through the application of molecular and genetic approaches. Starting from the basis of molecular markers and marker-assisted selection, these methods are becoming increasingly common in crop breeding. Whether it be plant genotyping focused on individual genes in an experiment or on thousands of genes simultaneously in microarray, these methods are integral to the progression of modern plant breeding programs. Crosses and segregations can be used to generate various types of doubled haploid and recombinant inbred lines, but genome-wide association mapping presents a powerful tool for comparative molecular-genetic analysis without hybridization. Expression of the identified genes can also be studied in individual analyses or through RNA-seq methods; these tools are very informative for plant breeding. An important component of modern plant breeding deals with proteomics and other biomolecules. Analyses of individual polypeptides and screens comparing the different profiles of a wide variety of proteins improve our knowledge of the molecular-genetic basis of plant biology as applied to crop breeding.

This Special Issue shows the current status of our understanding and research in the molecular-genetic basis of plant breeding. Eleven published papers address the scope, covering a wide and diverse breadth of modern technologies, scientific approaches, and research to better understand all aspects of the modern plant breeding of crops, including both native and commercially important plant species.

For example, a molecular and genetic analysis of downy mildew susceptibility in grapevine ended in a perfect conclusion, with the development of a new genetic resource for breeding based on genomics with the potential for further application in gene editing [1]. One year later, this research was extended through another published paper, wherein the *DMR6-1* gene was identified as the most promising candidate gene [2]. Another disease with *Verticillium* wilt in cotton told a different story, wherein RING-DUF1117 e3 ubiquitin ligase genes were successfully identified and characterized with important practical applications in cotton breeding [3]. Interestingly, a similar successful story was present in another paper [4], wherein genetic control of another type of *Fusarium* wilt in cucumber was identified using miRNA and transcriptome profiles.

However, abiotic stresses such as drought are also in the focus of molecular-genetic study for plant breeding. For example, methyleugenoln is a very important chemical compound for insect behavior and pollination. Therefore, its biosynthesis was studied using transcriptome analysis in wild ginger (*Asarum sieboldii*) with practical use in the breeding of the species [5]. In other papers, an important vernalization gene in wheat, *Vrn-B3* [6], and a flowering time gene in roses [7] were carefully and comprehensively studied both for academic knowledge and practical breeding.

To address current demands in wheat breeding, photo-thermo sensitive genic male sterility was described in a very timely and important published paper [8]. However, of particular interest to rice breeders will be reference [9], where authors made an ‘in-depth’ study of candidate genes involved in the very important trait of panicle grain number in rice. This study was based on an enormous research effort that combined Bulked segregants analysis, RNA-seq, and metabolomics. The theoretical background was thoroughly studied in the model *Arabidopsis* species, where *BIG3* and *BIG5* genes redundantly mediated vesicle trafficking transport of various substances within cells [10] while *cis*-regulatory regions of some duplicated genes resulted in very important methylation patterns of flavonoid biosynthesis genes in bread wheat [11]. Therefore, this Special Issue represents the excellent achievements of molecular genetic analyses in plants which can and certainly will be used in practical plant breeding.
